# Biological variation, reference change values and index of individuality of GDF-15

**DOI:** 10.1515/cclm-2021-0769

**Published:** 2022-03-28

**Authors:** Cindhya Sithiravel, Ragnhild Røysland, Bashir Alaour, Marit Sverresdotter Sylte, Janniche Torsvik, Heidi Strand, Michael Marber, Torbjørn Omland, Kristin Moberg Aakre

**Affiliations:** Multidisciplinary Laboratory Medicine and Medical Biochemistry, Akershus University Hospital, Lørenskog, Norway; Institute of Clinical Medicine, University of Oslo, Oslo, Norway; School of Cardiovascular Medicine and Sciences, King’s College London, London, UK; Department of Medical Biochemistry and Pharmacology, Haukeland University Hospital, Bergen, Norway; Department of Cardiology, Division of Medicine, Akershus University Hospital, Lørenskog, Norway; Department of Heart Disease, Haukeland University Hospital, Bergen, Norway; Department of Clinical Science, University of Bergen, Bergen, Norway

**Keywords:** analytical performance goal, biomarker, clinical interpretation, diagnosis, monitoring, prognosis

To the Editor,

GDF-15 is an upcoming and promising prognostic inflammatory biomarker found to be elevated in a wide range of diseases, including cancer and cardiovascular diseases [1], [2], [3]. Knowledge about the biological variation of GDF-15 is useful for suggesting analytical performance specifications. Furthermore, it aids in determining the number and frequency of samplings needed, as components with large biological variation may require several measurements to establish an individuals` homeostatic set point, which is necessary to confirm a diagnosis or assess prognosis. Reference change values (RCV) help determine whether the changes in results on serial testing within the same individual is clinically significant or can be explained by analytical or physiological variation. The index of individuality (II) allows us to distinguish between analyses where the population-based reference intervals or absolute cut-offs are relevant (index >1.4) and analyses where the subject-based reference intervals (i.e., delta changes) are more appropriate (index <0.6) [4, 5]. Previous studies have shown conflicting results concerning the biological variation, RCV and II of GDF-15 [6, 7].

We carried out a multicenter study at Haukeland University Hospital, Akershus University Hospital in Norway, King’s College London and Guy’s and St Thomas’ Hospital, United Kingdom. The study design was planned according to the EFLM checklist for biological variation studies (BIVAC) [8], and is described earlier [9]. Thirty healthy volunteers were recruited, 16 were women and two were daily smokers. Age range was 21–64 years (median 36 years). Participants had no evident disease, and no previous history of chronic illness. Screening tests including glucose, eGFR, high-sensitivity troponin and NT-proBNP were performed at baseline. Healthy-status was defined biochemically as:

– Non fasting glucose <7.8 mmol/L
– eGFR_(CKD-EPIcreat)_ >60 mL/min/1.73 m^2^
– cTnT <99th percentile for the assay (<14 ng/L)
– NT-proBNP <the local reference limit

Venous blood sampling was performed weekly, on the same weekday ±1 day, for 10 consecutive weeks, and separated serum was stored at −80 °C until analysis. GDF-15 was analyzed in duplicates, with the same reagent and calibrator lot at Akershus University Hospital on the Cobas e801 (Roche Diagnostics, Switzerland). The limit of detection (LOD) and limit of quantification (LOQ) was 400 ng/L (CV_A_ of ≤20%), and the measuring range was 400–20,000 ng/L. The CV_A_ was 0.47% at concentration 1,372–1,386 ng/L and 0.61% at concentration 7,373–7,458 ng/L.

**Figure 1:**
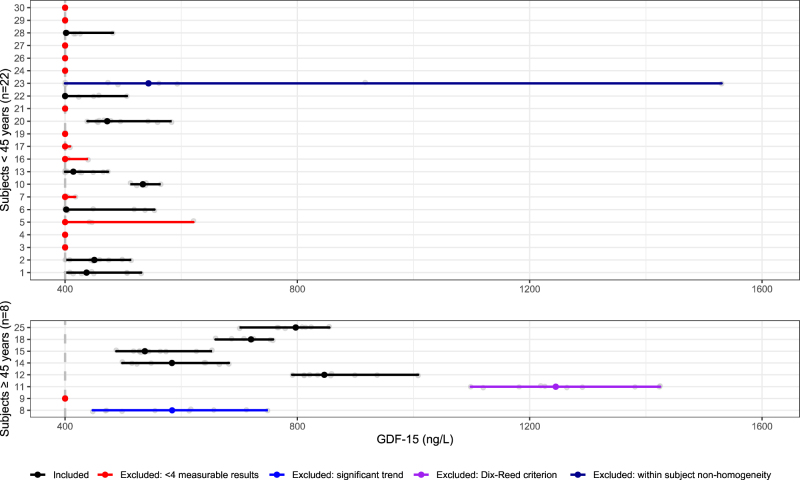
Median and range of 295 concentrations (ng/L) in the 30 participants. To visualize our data immeasurable values (values <LOD) are shown as 400 ng/L. These values are not included in the calculations of biological variation.

In total 17/30 (57%) individuals had ≥4 duplicate measurements above the LOD, and were eligible for further analysis. Analytical outliers were removed according to Burnett test, and outliers in mean values according to Reeds criteria [9]. The serial values for each individual were checked for linear trend using liner regression; subjects with significant trend (p-value of 0.01) were removed. Cochrane’s and Bartlett’s test were used to test homogeneity of analytical and within-subject variances, outliers were removed until homogeneity was achieved. Shapiro–Wilk test was used to test normality of the residuals. Thirteen subjects were included in the final analysis. Median concentrations were calculated for the total cohort, gender- and age-specific subgroups, differences in individual medians between age and gender groups were tested using Mann Whitney U test. Biological variation, RCV and II were calculated in the total cohort and age-stratified sub-groups (below 45 years and ≥45 years), as GDF-15 is higher in older individuals [3]. Age groups were chosen based on visual inspection of the data which fits well with the age-group stratification Krintus et al. have chosen (Figure 1) [6].

The distribution of the data was skewed (non-parametric) and calculations of σ_A_, σ_I_, σ_G_ were done on ln transformed data, using nested balanced ANOVA. The σ was thereafter retransformed into analytical variation (CV_A_), within-subject variation (CV_I_), and between-subject variation (CV_G_) using:

$${\text{CV}}_{\ln}=\sqrt{\left(\exp\;\sigma^{2}-1\right)\times 100}$$

in which σ is the estimated standard deviation for the ln-transformed data and CV_ln_ is the adjoining re-transformed CV.

The RCV values (with 95% confidence intervals) were calculated according to Fokkema et al. [10]:

$$\text{RCV pos}\left[\exp\left(1.96\text{x}2^{\frac{1}{2}}\times {\left(\sigma_{\text{A}}^{2}+\sigma_{\text{I}}^{2}\right)}^{\frac{1}{2}}\right)-1\right]\times 100$$

$$\text{RCV neg}=\left[\exp\left(-1.96\times 2^{\frac{1}{2}}\times {\left(\sigma_{\text{A}}^{2}+\sigma_{\text{I}}^{2}\right)}^{\frac{1}{2}}\right)-1\right]\times 100$$

in which σ_A_ is the analytic standard deviation and σ_I_ is the within-person standard deviation of the logarithmic data.

The II was calculated using the retransformed data as follows:

$$\text{II}=\frac{\sqrt{{\text{CV}}_{\text{A}}^{2}+{\text{CV}}_{\text{I}}^{2}}}{{\text{CV}}_{\text{G}}}$$

Desirable analytical performance goals was calculated as [5]:

$${\text{CV}}_{\text{A}}<\frac{1}{2}{\text{CV}}_{\text{I}}$$

$$\text{Bias}<\frac{1}{4}\sqrt{{\text{CV}}_{\text{I}}^{2}+{\text{CV}}_{\text{G}}^{2}}$$

Median GDF-15 concentration for all participants (13 individuals) was 515 (25 and 75 percentile, 463–652) ng/L. No difference in GDF-15 values between men (n=6) and women (n=7) was observed (median, 468 vs. 545 ng/L, for men vs. women, respectively; p-value for difference 0.36). Median concentration was higher in participants ≥45 years (n=8) compared to those <45 years (n=5): 719 vs. 470 ng/L, respectively; p-value for difference 0.03. The smokers were both excluded due to non-measurable (<LOD) results.

Calculated biological variation, RCV and II are presented in Table 1 together with number and reasons for participant exclusion. Results were similar across age groups except that CV_G_ was lower in younger subjects, and consequently the II was higher.

**Table 1:** Estimates of biological variation, reference change values (RCV) and index of individuality (II) for GDF-15, median concentration (25 and 75 percentile) are calculated after exclusion of outliers.

Estimation of biological variation, RCV and II (n=13)			
	Total cohort	Age <45 years	Age ≥45 years
Number of samples (participants)	120 (13)	52 (8)	49 (5)
Median concentrations (25–75 percentile), ng/L	515 (463–652)	470 (447–529)	719 (579–810)
CV_A_, mean (95% CI), %	1.8 (1.6–2.1)	1.9 (1.6–2.4)	1.6 (1.4–2.0)
CV_I_, mean (95% CI), %	7.6 (6.6–9.0)	7.4 (6.1–9.4)	7.9 (6.5–10.0)
CV_G_, mean (95% CI), %	23.6 (16.1–38.6)	6.5 (3.5–14.6)	19.2 (11.1–58.9)
RCV_pos_, mean (95% CI), %	24.3 (20.9–28.9)	23.6 (19.3–30.5)	24.9 (20.3–32.3)
RCV_neg_, mean (95% CI), %	−19.5 (−22.4 to −17.3)	−19.1 (−23.4 to −16.1)	−20.0 (−24.4 to −16.9)
II, mean (95% CI)	0.35 (0.20–0.51)	1.15 (0.40–2.03)	0.43 (0.11–0.79)

**Exclusion of samples or participants**			

Less than four samplings showed measurable results	14 participants (ID 3, 4, 5, 7, 9, 16, 17, 19, 21, 24, 26, 27, 29, 30)	13 participants (ID 3, 4, 5, 7, 16, 17, 19, 21, 24, 26, 27, 29, 30)	One participants (ID 9)
Analytical outliers	One sample result (ID 11)		One sample result (ID 11)
Exclusion due to a significant 10 week trend (p-value <0.01)	One participant (ID 8)		One participant (ID 8)
Exclusion due to Dix-Reed criterion	One participant (ID 11)		One participant (ID 11)
Exclusion due to within-subject non-homogeneity according to Bartlett or Cochrans test	One participant (ID 23)	One participant (ID 23)	
Exclusion due to analytical non-homogeneity according to Bartlett or Cochrans test	None	None	None

The lower part of the table shows number and reasons for sample or participant exclusions. RCV, reference change values; II, index of individuality.

Our study population and analysis method is similar to Krintus et al. We obtain similar estimates for biological variation, RCV, II and CV_A_ [6], confirming that results are robust. The observation that CV_G_ and II as a trend might be age-dependent is in line with the current interpretation of GDF-15 as a marker of biological age. However, due to overlapping CI of CV_G_ our results cannot be used to draw a firm conclusion of a significant larger inter-individual biological variation in older individuals [3]. It is noteworthy that 1/3 of our study population had GDF-15 levels below the LOD. This was also reported by Krintus, were 10/26 participants showed GDF-15 measurements below the LOD [6]. Meijers et al. [7] analyzed GDF-15 by a quantitative sandwich enzyme immunoassay technique (Quantikine^®^; R&D Systems, Inc., Minneapolis, MN, USA). For healthy subjects, they reported a higher CV_I_ (18.9%) and CV_A_ (15.2%), compared to our findings.

Based on our data we suggest the following analytical performance specifications; 3.8% as desirable CV_A_, and 6.2% as desirable analytical bias. In agreement with our and Krintus’ [6] results and the precision data reported by Roche, routine laboratories should be able to fulfill these performance specifications, utilizing the Roche Elecsys immunoassay.

The strength of our study is the standardized protocol and firm data analysis. The limitation is the relative low number of included subjects since many participants were excluded due to measured results below LOD, hence the variability in subjects with GDF-15 below LOD is unknown. The age groups were chosen arbitrary, and small group size resulted in larger uncertainty for the age-specific estimates, and these need confirmation in other studies. Also, our study only included healthy subjects, so the biological variation data are not applicable for interpreting clinical relevant changes in patients with chronic disease. However, Meijers et al. measured CV_I_ and CV_G_ in healthy subjects and heart failure patients, and did not find discrepancy between the two groups. Applicability in diseased populations still needs confirmation in future studies.

Our results confirm earlier findings of biological variation, RCV and II of GDF-15. The low biological variation and RCV indicate a biomarker useful for prognostication in individual subjects. The low II signals that subject-based reference intervals could be more appropriate than population based reference intervals or using an absolute cut offs (i.e., 1,800 ng/L) as suggested for prognostication today [3]. Finally, the biological variation is sufficiently large compared to the analytical variation, indicating that desirable analytical performance of GDF-15 measurements should be achievable in routine laboratories.
